# Midwifery-led care can lower caesarean section rates according to the Robson ten group classification system

**DOI:** 10.18332/ejm/119164

**Published:** 2020-03-31

**Authors:** Margaret Hanahoe

**Affiliations:** 1 National Maternity Hospital, Dublin, Ireland

**Keywords:** caesarean section, audit, community midwifery, Robson ten group classification system, low-risk nulliparous women

## Abstract

**INTRODUCTION:**

Midwifery-led care is recognised as the best choice of maternity care for low-risk women. Robson’s Ten Group Classification System (TGCS) is an internationally recognised audit tool, however there is no midwifery-led service presenting their statistics in this way. The objective of this study was to analyse caesarean section rates for the women attending midwifery-led care at the National Maternity Hospital Dublin, Ireland, using the Robson TGCS.

**METHODS:**

This is a retrospective study of electronic records for a total of 1097 women who were booked to attend the community midwife team in the National Maternity Hospital, during 2016 and 2017.

**RESULTS:**

The rate of caesarean section in low-risk nulliparous women (Robson Group 1) was under 6%, without affecting the perinatal outcome. The induction rate in nulliparous women (Group 2) was 36%, less than the national average the cesarean rates were quadruple in this group.

**CONCLUSIONS:**

Low-risk women who attend midwifery-led services, have a low caesarean section rate in this study. This was achieved with continuity of care, good antenatal preparation, and support throughout labour and birth by a dedicated team of midwives. Outcomes can only be truly compared if we use the same criteria to measure them. The TGCS demonstrates the effectiveness of midwifery-led care.

## INTRODUCTION

Although childbirth is not without risk, most women will have a safe birth and a healthy baby^1^. Over the last 20 years, the caesarean section (C/S) rate has increased worldwide^2^, leading to severe maternal complications including placenta accrete and placenta percreta.

The majority of the 62053 women giving birth in Ireland^3^, attend obstetric-led care for birth. However, midwives provide the care for women in labour and assist at a normal birth. Midwifery-led care has been defined as care where a midwife, in partnership with the woman, is develops a relationship of trust. They are the lead professional with responsibility for assessment of her needs, planning her care, providing continuity of care, making referrals to other professionals as appropriate, and ensuring provision of maternity services^4,5^. There is no doubt that some women's pregnancies and births have become more complicated due to many reasons including maternal age, fertility treatment, and obesity^6^, but the majority of pregnant women are still deemed to be low-risk and should have a straightforward birth under the care of a midwife^7^.

Fawsitt et al.^8^ have confirmed that midwifery-led care is very cost-effective, as it reduces labour costs and reduces medical intervention for women^9^. Fetal outcomes including preterm labour, neonatal death and admission to intensive care are lower for women attending midwifery-led care^4^.

The first Community Midwifery Service (CMS) was established in Ireland in 1999, at The National Maternity Hospital, Dublin, Ireland. The service is led and managed by a team of 14 midwives and is divided into two geographical locations. Women who attend midwifery-led services have continuity of care and a choice of homebirth or hospital birth. A room designated to the community midwives in the hospital labour ward offers a home-from-home atmosphere for women choosing to give birth in hospital. The team of midwives cares for women from their booking visit at approximately twelve weeks gestation up to seven days after birth. Clinics take place in 5 urban and rural locations, up to 30 km from the hospital. This includes a first booking visit, antenatal visits, antenatal classes, labour, and birth. Postnatal care takes place in the woman’s home. The low intervention labour policy was developed by the midwives, based on the NICE guidelines revised in 2014^10^. Antenatal classes are offered to all women, with particular emphasis on first-time mothers. The classes are honest, promote active birth, use hypnobirthing techniques, use visualization, include a visit to the labour room and may have a birth story from a mother who has given birth. The labour hopscotch^11^ is taught and used to encourage active labour. Nulliparous women are visited at home when contractions commence to encourage them to remain out of the hospital until labour is established.

However, the midwives must also adhere to hospital policy in relation to monitoring and induction of labour. The Community Midwifery birthing suite is not a standalone unit so if a woman requests an epidural, has been induced or requires monitoring, she remains in the birthing room with the community midwife and any further treatment, which may be deemed necessary for the woman, can be carried out there. Two midwives attend all hospital and homebirths. All assessments and any procedures are carried out by the Community Midwives while they offer one-to-one care throughout labour and birth. Women remain with the CMS for care, even if they have developed complications in pregnancy. A consultant obstetrician assists the scheme by reviewing women who are reffered by midwives, with concern in pregnancy. A senior registar is available to them for emergency consultations.

Prior to 2015, there was no standard measurement tool that could inform maternity-care health professionals in which group of women the rise of a caesarean section rates were occurring. The Robson’s Ten Group Classification System (TGCS) was recommended by the World Health Organisation (WHO) in 2015 to classify caesarean section. The time is right for midwives to also consider using the TGCS to assist audit in the reporting of midwifery-led care.

This study aims to examine the rates of C/S for women attending the Community Midwifery Services, by using the TGCS. This retrospective study applies a clinical audit tool to review the cases of 1097 pregnant women attending Community Midwifery Services in a Tertiary Maternity Care hospital during 2016 and 2017. Information already collected from all women who booked into the Community Midwives Service (CMS) was used anonymously to create a Report Table using TGCS (2001).

## METHODS

### Setting and participants

This is a retrospective study of electronic records for a total of 1097 women who were booked to attend the community midwife team in the National Maternity Hospital during 2016 and 2017. Women were accepted in the low-risk scheme using a simple inclusion\exclusion criterion: ‘Women who have no medical/surgical history that might affect pregnancy’^10^. This included no previous caesarean section, no fertility treatment, and a body mass index (BMI, kg/m^2^) under 35 for hospital birth or 30 for homebirth. Women were advised that the community midwives promote active labour and birth. A total of 1102 women booked with the CMS over the two years. There were five women not included in the study as they had developed diabetes or had a twin pregnancy diagnosed after the initial booking visit with the midwives at 12–14 weeks pregnant. It is important to emphasize that all the remaining women were included in the study even if complications developed in the pregnancy. In this study, there were no ethical dilemmas as the study did not involve intervention or manipulation of participants. The electronic information used in this study, was previously in existance and the data were collated to create this research. Ethical approval was obtained from the National Maternity Hospital Ethics Committee on 8 January 2018 and an Ethical exemption was also obtained from the UCD Ethics Committee in March 2018. No charges were requested by the NMH ethics Board.

### The Robson Ten Group Classification System

The TGCS is a standardized objective classification system where events and outcomes of labour and delivery can be incorporated^12^. Since 2015, Robson’s (2001) TGCS has been the internationally recognized tool for the Classification of Caesarean Section by the World Health Organisation (WHO); the WHO statement on the caesarean section^13^ notes: ‘… Let's start to collect data uniformly so that in the near future we will be able to move our focus from C/S rate at the population level to monitoring and discussing C/S rates and outcomes in each group of the Robson classification …’.

In 2011, Torloni et al.^14^ reported that using an internationally applicable C/S classification would facilitate auditing, analysing and comparing C/S rates across different settings globally.

This classification system is not just the study of C/S but is a platform for other research. Robson and the WHO recommend that any research being carried out in relation to labour or birth should be presented in a 10-group format that will allow for easier comparison, nationally and worldwide. The TGCS divides all women into ten basic groups according to parity, gestation, fetal presentation, and whether labour was induced or was spontaneous. All women fall into one of the ten groups, which is totally inclusive, mutually exclusive.

The TGCS involves the use of a simple table to gain greater understanding of clinical practice and assists in the comparison and contrast of clinical outcomes. The classification is not a criticism of practice but a data collection tool to count outcomes and improve the quality of care^15^.

Data were collected including the electronic charts of women. The participants needed to be identified as attending the community midwives. The data input was examined and cross-checked using the registration documents collected at each woman’s booking visit to ensure all women were recorded. Using this information, all women identified were placed in one of the ten groups, according to parity, gestation, fetal presentation and induced or spontaneous labour to produce the TGCS table. Community midwives do not take care of women with previous C/S. Therefore, these women (corresponding to Group 5) are omitted, and women with a multiple pregnancy (Group 8) are cared for by the fetal assessment unit and leave the care of the community midwives. An excel spreadsheet was developed to allow any non-computerized maternity unit to collect similar data.

## RESULTS

Table 1 presents the results of the TGCS for the Community Midwives Service for 2016 and 2017, including 8 nulliparous and 67 multiparous homebirths. The overall C/S rate in CMS in 2016 and 2017 was 107 (9.75%), and 37% of the women who attended the CMS over the 2 years were nulliparous. In all, 251 (64%) nulliparous women were in spontaneous labour and the C/S rate was 5.97%. This compares to 8.4% in the NMH over the same 2-year period^16,17^. Therefore, a woman in spontaneous labour at term with her first baby in a head-down position, had a 94.1% change of a vaginal birth with the community midwives.

**Table 1:** Ten Group Classification System for the Community Midwives Service for 2016 and 2017 (Total cases=1097; C/S cases=107; Total C/S rate=9.75%)

*Classification Group*	*Fraction with C/S in group*	*Fraction of group to total cases %*	*C/S rate within group %*	*Contribution of each group to total C/S rate %*
**1** Nulliparous single cephalic ≥37weeks spontaneous labour	15/251	22.88	5.97	1.36
**2a** Nulliparous single cephalic ≥37weeks induced **2b** C/S before labour	40/144 4/4	13.49	30	4.0
**3** Multiparous (excluding previous caesarian sections) single cephalic ≥37weeks spontaneous labour	4/549	50.04	0.72	0.4
**4a** Multiparous single cephalic ≥37weeks induced **4b** C/S before labour	4/94 5/5	9.02	9.1	0.82
**5** Previous caesarean section single cephalic ≥37 weeks	0	0	0	0
**6** All nulliparous breeches	17/17	1.55	100	1.56
**7** All multiparous breeches (including previous caesarean sections)	12/13	1.19	92.3	1.01
**8** All multiple pregnancies (including previous caesarean sections)	0	0	0	0
**9** All abnormal lies (including previous caesarean sections)	3/3	0.27	100	0.3
**10** All single cephalic ≤36weeks (including previous sections)	3/17	1.55	17.6	0.3

It is very evident that the C/S rate rose rapidly to 30% if a nulliparous woman had an induced labour or pre-labour C/S. This group of women also contributes the most substantial proportion of women having C/S, in fact 4% of the total 9.75% C/S rate. Four women had a pre-labour C/S for a placenta previa or PET^17^. Women attending the Community Midwives Services strive to have normal labour but the midwifery team must adhere to criteria for induction within the hospital. The induction rate for first-time mothers was 36%, compared to the hospital rate of 43.4%.

The largest group of women cared for by the community midwives had a previous vaginal birth and were in spontaneous labour. These women had the best outcomes with a very low C/S rate of 0.72%. These women had a high homebirth success rate, with 85% achieving a homebirth. The induction rate was 14.6% for multiparous women and the C/S rate in this group was 4.2%. The C/S rate of 9.1% in this group includes the 5 women who had a pre-labour C/S for placenta previa, IUGR or a previous 3rd degree tear. Breech C/S rate is high as all women who were diagnosed with a breech presentation were booked for an elective C/S as recommended by the Term Breech Trial^18^. One woman achieved a vaginal breech birth. Although most women have an elective C/S in this group, it still only contributes 2.57% to the total C/S rate.

Women giving birth with an abnormal lie over 37 weeks is a small group with only 3 women recorded. Globally, this figure is 0.3–0.5% or 3–5 per 1000 births. Robson^12^ refers to this group as the ‘quality assurance’ group as the C/S rate should always be 100% and the occurrence is always similar. Preterm labour had a relatively high C/S rate of 12.5% as women usually present with a pregnancy induced problem such as pre-eclampsia or fetal compromise. In line with the Sandall et al.^4^ study, this group of women was unexplainably small at 1.5% compared with the hospital preterm rate of 4.3%.

In this study, further research was completed on Group 1 (low-risk nulliparous women). One infant had an apgar score <7 (0.4%) and 26 babies (10.3%) were admitted to the neonatal unit. In the same group, 18.5% of the hospital babies were admitted to the neonatal unit.

## DISCUSSION

Normal-risk women should be encouraged to attend a midwife for their maternity care. There is very strong evidence that better continuity of care and provider of care lead to better outcomes for the mother and baby^19^. According to Dabrowski^20^, when the woman has a relationship with her midwife there is greater trust and the woman is more likely to share information with the midwife that could be important in her care and pregnancy.

Cochrane Review into Midwifery-led Care^21^, shows that care and safety of mothers and newborn babies should be at the very heart of maternity services. Women who received continued care throughout pregnancy and birth from a small team of midwives were less likely to give birth prematurely and required fewer interventions during labour and birth. Brocklehurst et al.^22^ reviewed 64538 eligible women showing fewer interventions with no impact on perinatal outcomes.

The findings of the current study show that the necessary data collection and application of the Robson TGCS can be carried out quite simply and effectively, and in a range of settings.

The increasing C/S rate worldwide has led to an increase in maternal mortality^23^. The ultimate aim of any clinical audit should lead to improvements in women’s care^24^. The use of audit helps raise the quality of healthcare, and highlights the most essential concern of any health professional: to optimize clinical performance and provide the best possible care for women. Maternity care providers can give better care with better data^25^. The examination of the data collected by the CMS in the current study can help improve quality of care by examining and discussing the C/S carried out in each group. While every service produces data that can be analysed, the current study findings would support the view that all professionals offering maternity care should use the same tool, the TGCS, as recommended by the WHO^26^. The TGCS tables from the community midwifery service can then be compared with those of other services, hospitals and countries around the world.

### Strengths and limitations

This is the first study to examine C/S rates in a Community Midwifery setting by applying the WHO^26^ recommended TGCS tool. On the other hand, the use of a pre-determined structured data set means that the level of detailed data is a limiting factor. That said, the use of the TGCS as part of a clinical audit offers a basic framework for interesting research. It must be commented that the women who attended the community midwifery service had chosen that model of care and may have sought a low-intervention birth.

## CONCLUSIONS

The TGCS is a starting point to review many areas of research for midwives. Community Midwifery Services can demonstrate reductions in interventions in labour for low-risk women without affecting maternal or fetal outcomes. These statistics when delivered appropriately can help women make the choice between maternity services based on the outcomes of labour.

This study successfully demonstrates the effectiveness of midwifery-led care in maintaining a reasonable C/S rate for low-risk women without affecting fetal or maternal outcomes. The use of the Robson Ten Group Classification System^27,28^ assists audit by allowing a comparison and analysis of similar groups of women availing themselves of midwifery-led care. Over time, this system can be used and shared with units around the world. This quality improvement initiative may help us learn from other midwifery-led practices how to maintain or reduce interventions and C/S rates. Midwives need to focus on the care of low-risk, first-time mothers, as reducing the C/S rate in this group will influence C/S rates in the future.

## References

[1] Betrán AP, Temmerman M, Kingdon C (2018). Interventions to reduce unnecessary caesarean sections in healthy women and babies. Lancet.

[2] Haas DM, Morgan S, Contreras K, Enders S (2018). Vaginal preparation with antiseptic solution before cesarean section for preventing postoperative infections. Cochrane Database Syst Rev.

[3] Central Statistics Office Births, Deaths and Marriages.

[4] Sandall J, Soltani H, Gates S, Shennan A, Devane D (2015). Midwife-led continuity models versus other models of care for childbearing women. Cochrane Database Syst Rev.

[5] Royal College of Obstetricians and Gynaecologists Safer Childbirth: Minimum standards for the organization and delivery of care in labour.

[6] Lamminpää R, Vehviläinen-Julkunen K, Gissler M, Selander T, Heinonen S (2016). Pregnancy outcomes of overweight and obese women aged 35 years or older–A registry-based study in Finland. Obes Res Clin Pract.

[7] Walsh D, Devane D (2012). A metasynthesis of midwife-led care. Qual Health Res.

[8] Fawsitt CG, Bourke J, Murphy A, McElroy B, Lutomski JE, Murphy R, Greene RA (2017). A Cost-Benefit Analysis of Two Alternative Models of Maternity Care in Ireland. Appl Health Econ Health Policy.

[9] Kenny C, Devane D, Normand C, Clarke M, Howard A, Begley C (2015). A cost-comparison of midwife-led compared with consultant-led maternity care in Ireland (the MidU study). Midwifery.

[10] National Institute for Health and Care Excellence Addendum to Clinical Guideline CG190, Intrapartum care for healthy women and babies. Clinical Guideline Addendum 190.2: Methods, evidence and recommendations.

[11] Thompson S, Coughlan B, Doherty J (2020). An Evaluation of the Labour Hopscotch Framework at the National Maternity Hospital: Findings and Recommendations [Video].

[12] Rossen J, Lucovnik M, Eggebø TM, Tul N, Murphy M, Vistad I, Robson M (2017). A method to assess obstetric outcomes using the 10-Group Classification System: a quantitative descriptive study. BMJ Open.

[13] Betran AP, Torloni MR, Zhang JJ, Gülmezoglu AM (2015). WHO Statement on caesarean section rates.

[14] Torloni MR, Betran AP, Souza JP, Widmer M, Allen T, Gulmezoglu M, Merialdi M (2011). Classifications for cesarean section: a systematic review. PloS One.

[15] Robson MS (2018). The 10-Group Classification System–a new way of thinking. Am J Obstet Gynecol.

[16] National Maternity Hospital (2016). Community Midwifery Guidelines: Clinical report.

[17] National Maternity Hospital (2017). Community Midwives Guidelines: Clinical report.

[18] Hannah ME, Hannah WJ, Hewson SA, Hodnett ED, Saigal S, Willan AR (2000). Planned caesarean section versus planned vaginal birth for breech presentation at term: a randomised multicentre trial. Term Breech Trial Collaborative Group. Lancet.

[19] Smith AHK, Dixon AL, Page LA (2009). Health-care professionals' views about safety in maternity services: a qualitative study. Midwifery.

[20] Dabrowski R (2016). Amend to NICE Intrapartum Care Guideline.

[21] Sandall J, Devane D, Soltani H, Hatem M, Gates S (2010). Improving quality and safety in maternity care: the contribution of midwife-led care. J Midwifery Womens Health.

[22] Brocklehurst P, Hardy P, Hollowell J (2011). Perinatal and maternal outcomes by planned place of birth for healthy women with low risk pregnancies: the Birthplace in England national prospective cohort study. BMJ.

[23] Fahmy WM, Crispim CA, Cliffe S (2018). Association between maternal death and cesarean section in Latin America: a systematic literature review. Midwifery.

[24] Ronsmans C (2001). What is the evidence for the role of audits to improve the quality of obstetric care. Safe motherhood strategies: a review of the Evidence. Studies in HSO&P.

[25] Neville G, Robson M (2001). Current progress in Obstetrics & Gynaecology. Caesarean Section Rates: Much Ado about Nothing or a Marker of Quality Care?.

[26] World Health Organization (2015). WHO statement on caesarean section rates (No. WHO/RHR/15.02).

[27] Robson MS (2001). Can we reduce the caesarean section rate?. Best Pract Res Clin Obstet Gynaecol.

[28] Robson MS (2001). Classification of caesarean sections. Fetal and Maternal Medicine Review.

